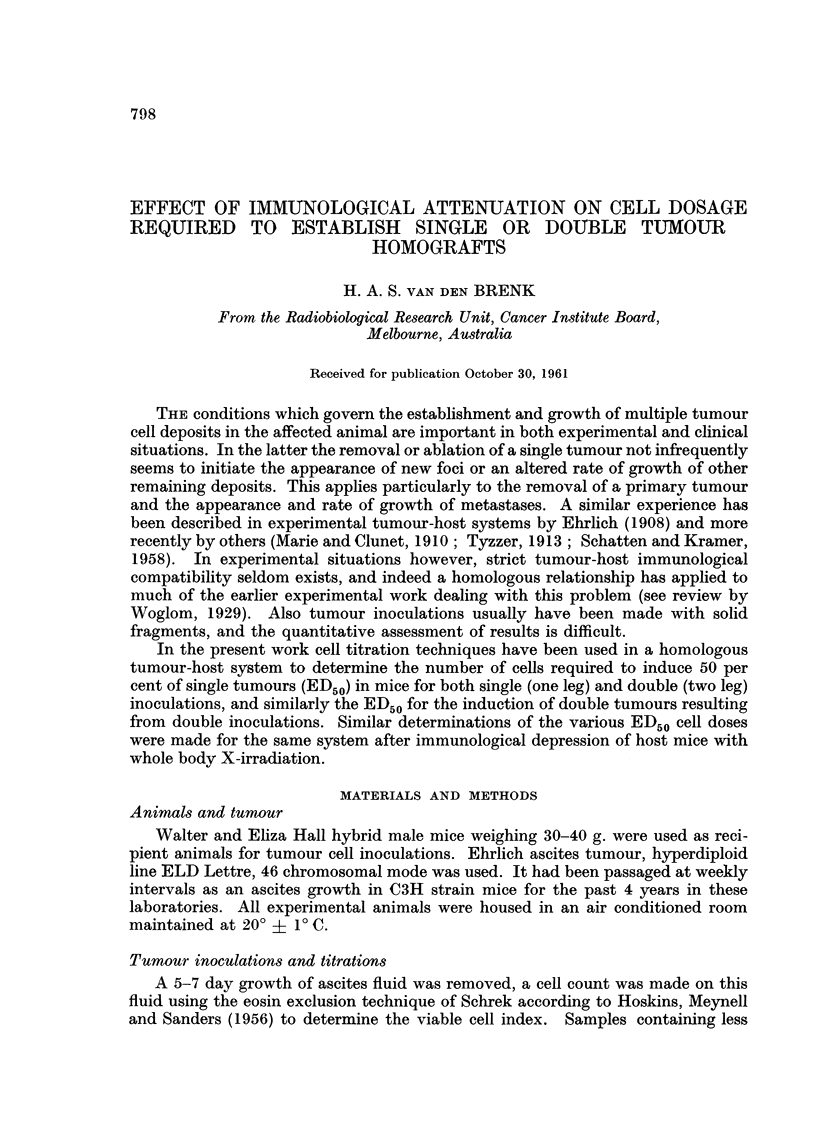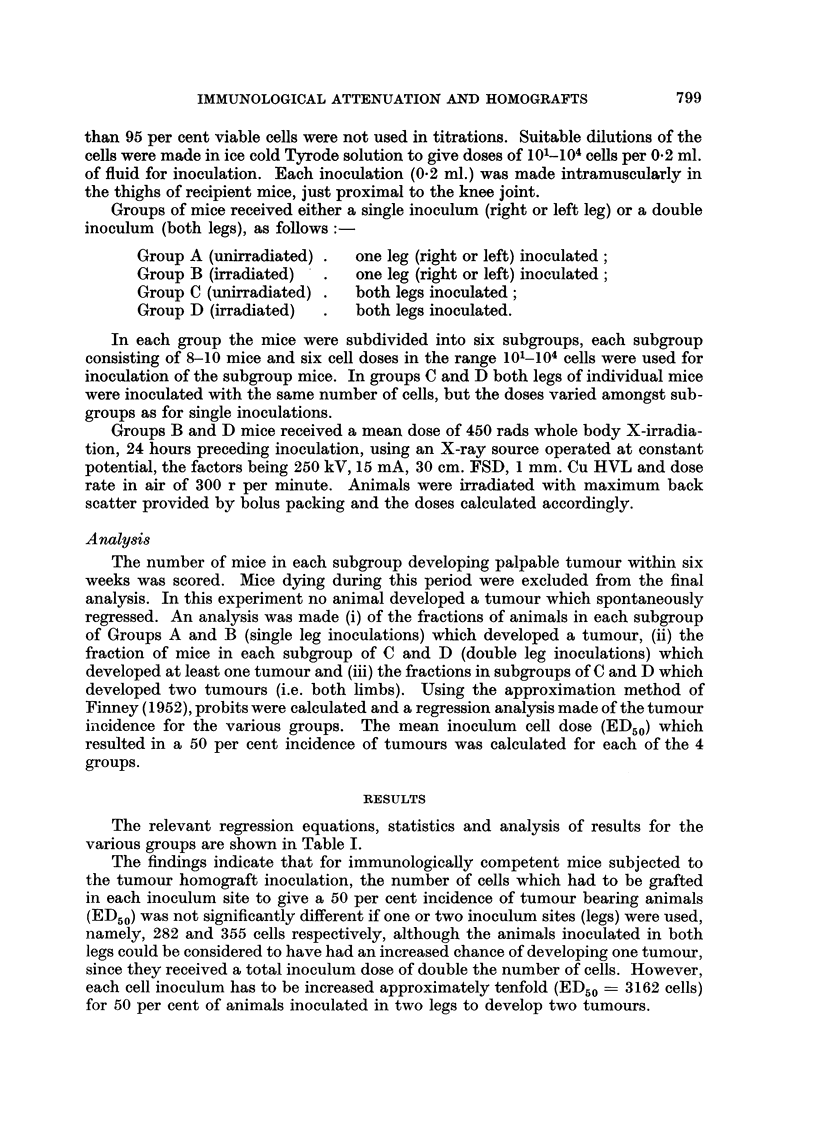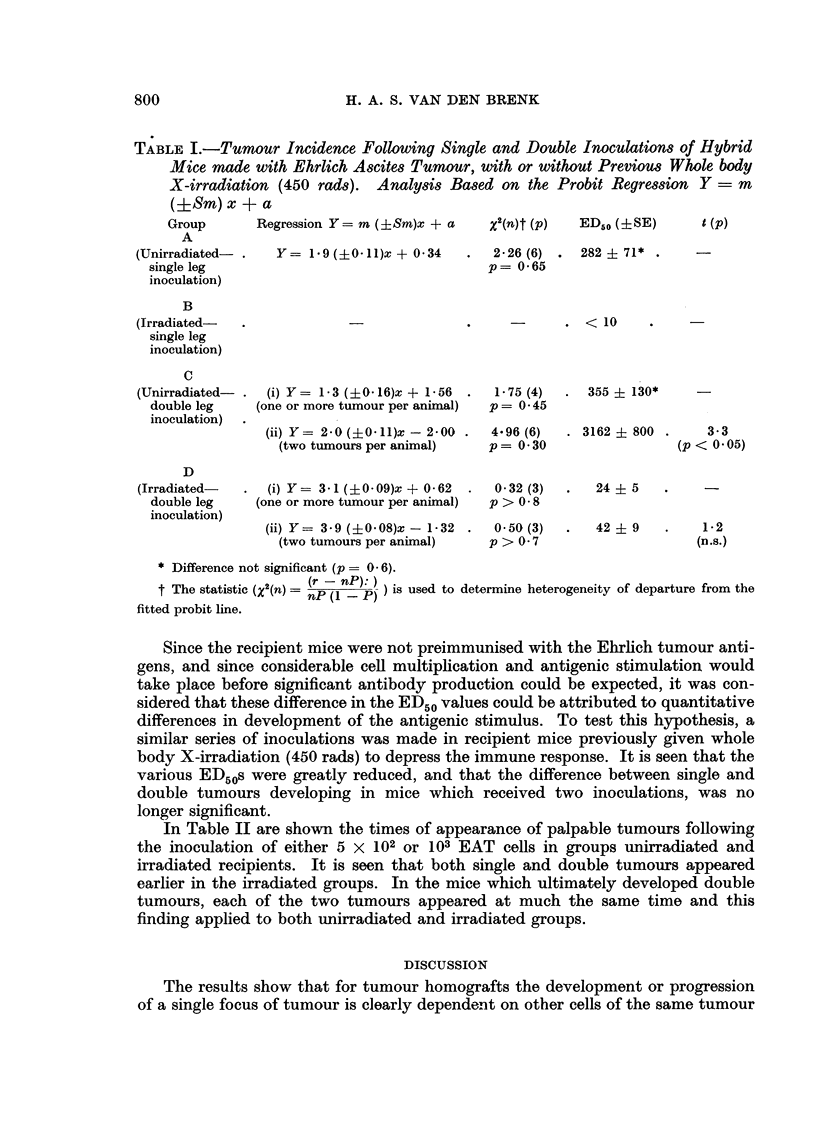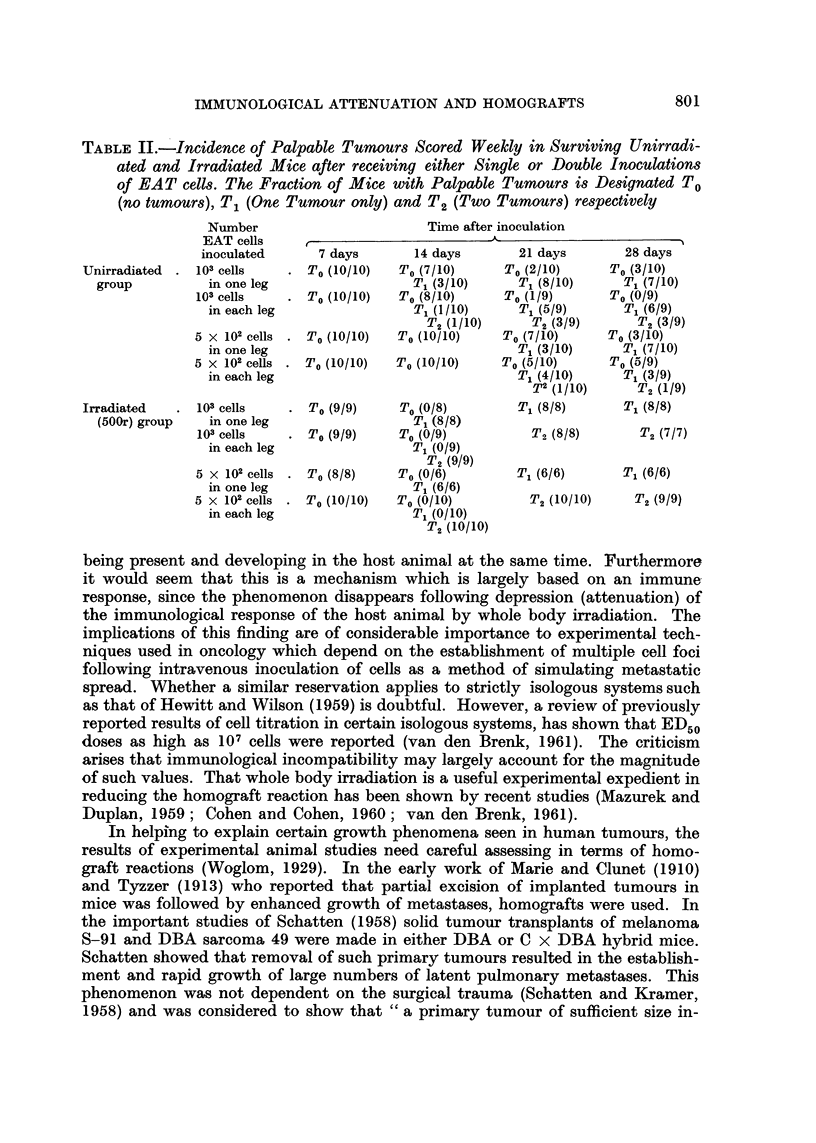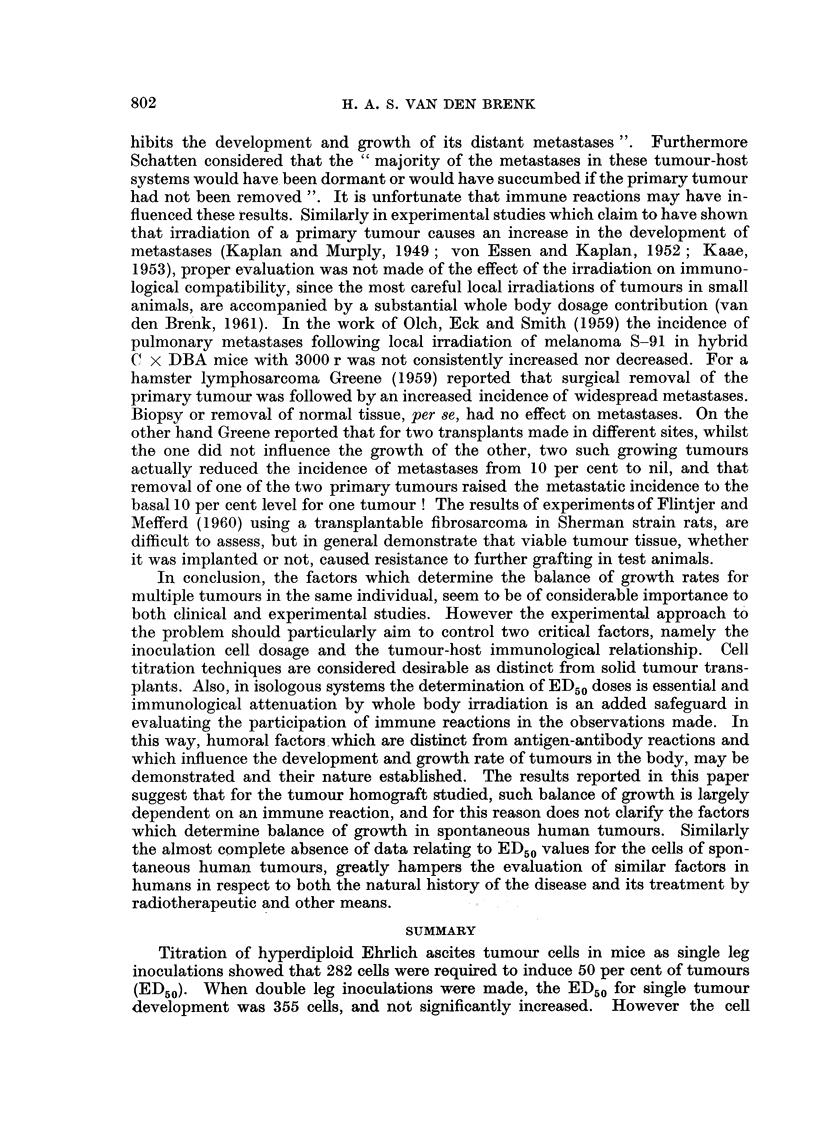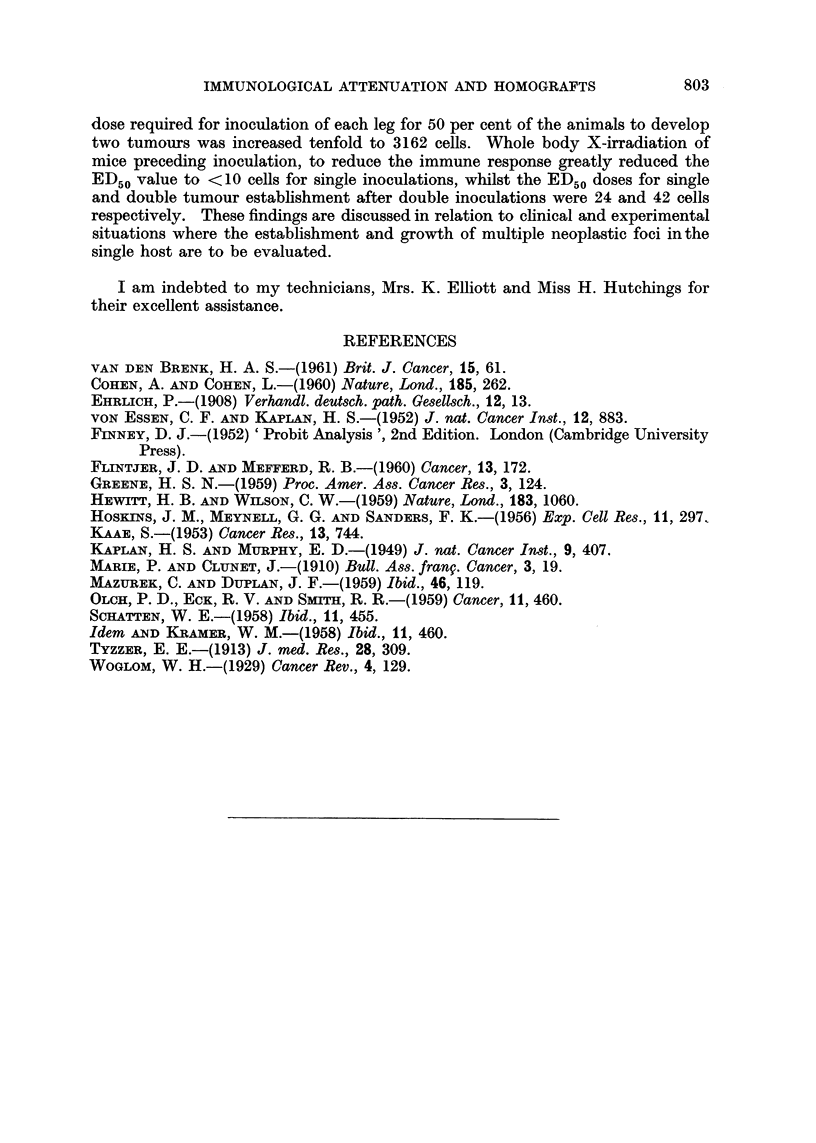# Effect of Immunological Attenuation on Cell Dosage Required to Establish Single or Double Tumour Homografts

**DOI:** 10.1038/bjc.1961.90

**Published:** 1961-12

**Authors:** H. A. S. van den Brenk


### EFFECT OF IMMUNOLOGICAL ATTENUATION ON CELL DOSAGE REQUIRED TO ESTABLISH SINGLE OR DOUBLE TUMOUR HOMOGRAFTS

H. A. S. VAN DEN BRENK

*From the Radiobiological Research Unit, Cancer Institute Board, Melbourne, Australia*

Received for publication October 30, 1961

THE conditions which govern the establishment and growth of multiple tumour cell deposits in the affected animal are important in both experimental and clinical situations. In the latter the removal or ablation of a single tumour not infrequently seems to initiate the appearance of new foci or an altered rate of growth of other remaining deposits. This applies particularly to the removal of a primary tumour and the appearance and rate of growth of metastases. A similar experience has been described in experimental tumour-host systems by Ehrlich (1908) and more recently by others (Marie and Clunet, 1910 ; Tyzzer, 1913 ; Schatten and Kramer, 1958). In experimental situations however, strict tumour-host immunological compatibility seldom exists, and indeed a homologous relationship has applied to much of the earlier experimental work dealing with this problem (see review by Woglom, 1929). Also tumour inoculations usually have been made with solid fragments, and the quantitative assessment of results is difficult.

In the present work cell titration techniques have been used in a homologous tumour-host system to determine the number of cells required to induce 50 per cent of single tumours ($ED_{50}$) in mice for both single (one leg) and double (two leg) inoculations, and similarly the $ED_{50}$ for the induction of double tumours resulting from double inoculations. Similar determinations of the various $ED_{50}$ cell doses were made for the same system after immunological depression of host mice with whole body X-irradiation.

### MATERIALS AND METHODS

### *Animals and tumour*

Walter and Eliza Hall hybrid male mice weighing 30–40 g. were used as recipient animals for tumour cell inoculations. Ehrlich ascites tumour, hyperdiploid line ELD Lettre, 46 chromosomal mode was used. It had been passaged at weekly intervals as an ascites growth in C3H strain mice for the past 4 years in these laboratories. All experimental animals were housed in an air conditioned room maintained at $20° \pm 1° C$.

### *Tumour inoculations and titrations*

A 5–7 day growth of ascites fluid was removed, a cell count was made on this fluid using the eosin exclusion technique of Schrek according to Hoskins, Meynell and Sanders (1956) to determine the viable cell index. Samples containing less than 95 per cent viable cells were not used in titrations. Suitable dilutions of the cells were made in ice cold Tyrode solution to give doses of $10^1$–$10^4$ cells per 0·2 ml. of fluid for inoculation. Each inoculation (0·2 ml.) was made intramuscularly in the thighs of recipient mice, just proximal to the knee joint.

Groups of mice received either a single inoculum (right or left leg) or a double inoculum (both legs), as follows :—

| | |
|---|---|
| Group A (unirradiated) . | one leg (right or left) inoculated ; |
| Group B (irradiated) . | one leg (right or left) inoculated ; |
| Group C (unirradiated) . | both legs inoculated ; |
| Group D (irradiated) . | both legs inoculated. |

In each group the mice were subdivided into six subgroups, each subgroup consisting of 8–10 mice and six cell doses in the range $10^1$–$10^4$ cells were used for inoculation of the subgroup mice. In groups C and D both legs of individual mice were inoculated with the same number of cells, but the doses varied amongst subgroups as for single inoculations.

Groups B and D mice received a mean dose of 450 rads whole body X-irradiation, 24 hours preceding inoculation, using an X-ray source operated at constant potential, the factors being 250 kV, 15 mA, 30 cm. FSD, 1 mm. Cu HVL and dose rate in air of 300 r per minute. Animals were irradiated with maximum back scatter provided by bolus packing and the doses calculated accordingly.

*Analysis*

The number of mice in each subgroup developing palpable tumour within six weeks was scored. Mice dying during this period were excluded from the final analysis. In this experiment no animal developed a tumour which spontaneously regressed. An analysis was made (i) of the fractions of animals in each subgroup of Groups A and B (single leg inoculations) which developed a tumour, (ii) the fraction of mice in each subgroup of C and D (double leg inoculations) which developed at least one tumour and (iii) the fractions in subgroups of C and D which developed two tumours (i.e. both limbs). Using the approximation method of Finney (1952), probits were calculated and a regression analysis made of the tumour incidence for the various groups. The mean inoculum cell dose ($ED_{50}$) which resulted in a 50 per cent incidence of tumours was calculated for each of the 4 groups.

RESULTS

The relevant regression equations, statistics and analysis of results for the various groups are shown in Table I.

The findings indicate that for immunologically competent mice subjected to the tumour homograft inoculation, the number of cells which had to be grafted in each inoculum site to give a 50 per cent incidence of tumour bearing animals ($ED_{50}$) was not significantly different if one or two inoculum sites (legs) were used, namely, 282 and 355 cells respectively, although the animals inoculated in both legs could be considered to have had an increased chance of developing one tumour, since they received a total inoculum dose of double the number of cells. However, each cell inoculum has to be increased approximately tenfold ($ED_{50}$ = 3162 cells) for 50 per cent of animals inoculated in two legs to develop two tumours.

TABLE I.—*Tumour Incidence Following Single and Double Inoculations of Hybrid Mice made with Ehrlich Ascites Tumour, with or without Previous Whole body X-irradiation* (450 *rads*). *Analysis Based on the Probit Regression* $Y = m\ (\pm Sm)\ x + a$

| Group | Regression $Y = m\ (\pm Sm)x + a$ | $\chi^2(n)$† $(p)$ | $ED_{50}$ ($\pm$SE) | $t\ (p)$ |
|---|---|---|---|---|
| A (Unirradiated—single leg inoculation) | $Y = 1{\cdot}9\ (\pm 0{\cdot}11)x + 0{\cdot}34$ | $2{\cdot}26$ (6) $p = 0{\cdot}65$ | $282 \pm 71$* | — |
| B (Irradiated—single leg inoculation) | — | — | $< 10$ | — |
| C (Unirradiated—double leg inoculation) | (i) $Y = 1{\cdot}3\ (\pm 0{\cdot}16)x + 1{\cdot}56$ (one or more tumour per animal) | $1{\cdot}75$ (4) $p = 0{\cdot}45$ | $355 \pm 130$* | — |
| | (ii) $Y = 2{\cdot}0\ (\pm 0{\cdot}11)x - 2{\cdot}00$ (two tumours per animal) | $4{\cdot}96$ (6) $p = 0{\cdot}30$ | $3162 \pm 800$ | $3{\cdot}3$ $(p < 0{\cdot}05)$ |
| D (Irradiated—double leg inoculation) | (i) $Y = 3{\cdot}1\ (\pm 0{\cdot}09)x + 0{\cdot}62$ (one or more tumour per animal) | $0{\cdot}32$ (3) $p > 0{\cdot}8$ | $24 \pm 5$ | — |
| | (ii) $Y = 3{\cdot}9\ (\pm 0{\cdot}08)x - 1{\cdot}32$ (two tumours per animal) | $0{\cdot}50$ (3) $p > 0{\cdot}7$ | $42 \pm 9$ | $1{\cdot}2$ (n.s.) |

* Difference not significant ($p = 0{\cdot}6$).

† The statistic ($\chi^2(n) = \frac{(r - nP)^2}{nP\,(1 - P)}$) is used to determine heterogeneity of departure from the fitted probit line.

Since the recipient mice were not preimmunised with the Ehrlich tumour antigens, and since considerable cell multiplication and antigenic stimulation would take place before significant antibody production could be expected, it was considered that these difference in the $ED_{50}$ values could be attributed to quantitative differences in development of the antigenic stimulus. To test this hypothesis, a similar series of inoculations was made in recipient mice previously given whole body X-irradiation (450 rads) to depress the immune response. It is seen that the various $ED_{50}$s were greatly reduced, and that the difference between single and double tumours developing in mice which received two inoculations, was no longer significant.

In Table II are shown the times of appearance of palpable tumours following the inoculation of either $5 \times 10^2$ or $10^3$ EAT cells in groups unirradiated and irradiated recipients. It is seen that both single and double tumours appeared earlier in the irradiated groups. In the mice which ultimately developed double tumours, each of the two tumours appeared at much the same time and this finding applied to both unirradiated and irradiated groups.

### DISCUSSION

The results show that for tumour homografts the development or progression of a single focus of tumour is clearly dependent on other cells of the same tumour

TABLE II.—*Incidence of Palpable Tumours Scored Weekly in Surviving Unirradiated and Irradiated Mice after receiving either Single or Double Inoculations of EAT cells. The Fraction of Mice with Palpable Tumours is Designated* $T_0$ *(no tumours),* $T_1$ *(One Tumour only) and* $T_2$ *(Two Tumours) respectively*

| | Number EAT cells inoculated | Time after inoculation: 7 days | 14 days | 21 days | 28 days |
|---|---|---|---|---|---|
| Unirradiated group | $10^3$ cells in one leg | $T_0$ (10/10) | $T_0$ (7/10) $T_1$ (3/10) | $T_0$ (2/10) $T_1$ (8/10) | $T_0$ (3/10) $T_1$ (7/10) |
| | $10^3$ cells in each leg | $T_0$ (10/10) | $T_0$ (8/10) $T_1$ (1/10) $T_2$ (1/10) | $T_0$ (1/9) $T_1$ (5/9) $T_2$ (3/9) | $T_0$ (0/9) $T_1$ (6/9) $T_2$ (3/9) |
| | $5 \times 10^2$ cells in one leg | $T_0$ (10/10) | $T_0$ (10/10) | $T_0$ (7/10) $T_1$ (3/10) | $T_0$ (3/10) $T_1$ (7/10) |
| | $5 \times 10^2$ cells in each leg | $T_0$ (10/10) | $T_0$ (10/10) | $T_0$ (5/10) $T_1$ (4/10) $T^2$ (1/10) | $T_0$ (5/9) $T_1$ (3/9) $T_2$ (1/9) |
| Irradiated (500r) group | $10^3$ cells in one leg | $T_0$ (9/9) | $T_0$ (0/8) $T_1$ (8/8) | $T_1$ (8/8) | $T_1$ (8/8) |
| | $10^3$ cells in each leg | $T_0$ (9/9) | $T_0$ (0/9) $T_1$ (0/9) $T_2$ (9/9) | $T_2$ (8/8) | $T_2$ (7/7) |
| | $5 \times 10^2$ cells in one leg | $T_0$ (8/8) | $T_0$ (0/6) $T_1$ (6/6) | $T_1$ (6/6) | $T_1$ (6/6) |
| | $5 \times 10^2$ cells in each leg | $T_0$ (10/10) | $T_0$ (0/10) $T_1$ (0/10) $T_2$ (10/10) | $T_2$ (10/10) | $T_2$ (9/9) |

being present and developing in the host animal at the same time. Furthermore it would seem that this is a mechanism which is largely based on an immune response, since the phenomenon disappears following depression (attenuation) of the immunological response of the host animal by whole body irradiation. The implications of this finding are of considerable importance to experimental techniques used in oncology which depend on the establishment of multiple cell foci following intravenous inoculation of cells as a method of simulating metastatic spread. Whether a similar reservation applies to strictly isologous systems such as that of Hewitt and Wilson (1959) is doubtful. However, a review of previously reported results of cell titration in certain isologous systems, has shown that $ED_{50}$ doses as high as $10^7$ cells were reported (van den Brenk, 1961). The criticism arises that immunological incompatibility may largely account for the magnitude of such values. That whole body irradiation is a useful experimental expedient in reducing the homograft reaction has been shown by recent studies (Mazurek and Duplan, 1959; Cohen and Cohen, 1960; van den Brenk, 1961).

In helping to explain certain growth phenomena seen in human tumours, the results of experimental animal studies need careful assessing in terms of homograft reactions (Woglom, 1929). In the early work of Marie and Clunet (1910) and Tyzzer (1913) who reported that partial excision of implanted tumours in mice was followed by enhanced growth of metastases, homografts were used. In the important studies of Schatten (1958) solid tumour transplants of melanoma S-91 and DBA sarcoma 49 were made in either DBA or C $\times$ DBA hybrid mice. Schatten showed that removal of such primary tumours resulted in the establishment and rapid growth of large numbers of latent pulmonary metastases. This phenomenon was not dependent on the surgical trauma (Schatten and Kramer, 1958) and was considered to show that " a primary tumour of sufficient size inhibits the development and growth of its distant metastases ". Furthermore Schatten considered that the " majority of the metastases in these tumour-host systems would have been dormant or would have succumbed if the primary tumour had not been removed ". It is unfortunate that immune reactions may have influenced these results. Similarly in experimental studies which claim to have shown that irradiation of a primary tumour causes an increase in the development of metastases (Kaplan and Murply, 1949 ; von Essen and Kaplan, 1952 ; Kaae, 1953), proper evaluation was not made of the effect of the irradiation on immunological compatibility, since the most careful local irradiations of tumours in small animals, are accompanied by a substantial whole body dosage contribution (van den Brenk, 1961). In the work of Olch, Eck and Smith (1959) the incidence of pulmonary metastases following local irradiation of melanoma S–91 in hybrid C × DBA mice with 3000 r was not consistently increased nor decreased. For a hamster lymphosarcoma Greene (1959) reported that surgical removal of the primary tumour was followed by an increased incidence of widespread metastases. Biopsy or removal of normal tissue, *per se*, had no effect on metastases. On the other hand Greene reported that for two transplants made in different sites, whilst the one did not influence the growth of the other, two such growing tumours actually reduced the incidence of metastases from 10 per cent to nil, and that removal of one of the two primary tumours raised the metastatic incidence to the basal 10 per cent level for one tumour ! The results of experiments of Flintjer and Mefferd (1960) using a transplantable fibrosarcoma in Sherman strain rats, are difficult to assess, but in general demonstrate that viable tumour tissue, whether it was implanted or not, caused resistance to further grafting in test animals.

In conclusion, the factors which determine the balance of growth rates for multiple tumours in the same individual, seem to be of considerable importance to both clinical and experimental studies. However the experimental approach to the problem should particularly aim to control two critical factors, namely the inoculation cell dosage and the tumour-host immunological relationship. Cell titration techniques are considered desirable as distinct from solid tumour transplants. Also, in isologous systems the determination of $ED_{50}$ doses is essential and immunological attenuation by whole body irradiation is an added safeguard in evaluating the participation of immune reactions in the observations made. In this way, humoral factors which are distinct from antigen-antibody reactions and which influence the development and growth rate of tumours in the body, may be demonstrated and their nature established. The results reported in this paper suggest that for the tumour homograft studied, such balance of growth is largely dependent on an immune reaction, and for this reason does not clarify the factors which determine balance of growth in spontaneous human tumours. Similarly the almost complete absence of data relating to $ED_{50}$ values for the cells of spontaneous human tumours, greatly hampers the evaluation of similar factors in humans in respect to both the natural history of the disease and its treatment by radiotherapeutic and other means.

### SUMMARY

Titration of hyperdiploid Ehrlich ascites tumour cells in mice as single leg inoculations showed that 282 cells were required to induce 50 per cent of tumours ($ED_{50}$). When double leg inoculations were made, the $ED_{50}$ for single tumour development was 355 cells, and not significantly increased. However the cell dose required for inoculation of each leg for 50 per cent of the animals to develop two tumours was increased tenfold to 3162 cells. Whole body X-irradiation of mice preceding inoculation, to reduce the immune response greatly reduced the $ED_{50}$ value to $<10$ cells for single inoculations, whilst the $ED_{50}$ doses for single and double tumour establishment after double inoculations were 24 and 42 cells respectively. These findings are discussed in relation to clinical and experimental situations where the establishment and growth of multiple neoplastic foci in the single host are to be evaluated.

I am indebted to my technicians, Mrs. K. Elliott and Miss H. Hutchings for their excellent assistance.

### REFERENCES

VAN DEN BRENK, H. A. S.—(1961) *Brit. J. Cancer*, **15**, 61.
COHEN, A. AND COHEN, L.—(1960) *Nature, Lond.*, **185**, 262.
EHRLICH, P.—(1908) *Verhandl. deutsch. path. Gesellsch.*, **12**, 13.
VON ESSEN, C. F. AND KAPLAN, H. S.—(1952) *J. nat. Cancer Inst.*, **12**, 883.
FINNEY, D. J.—(1952) 'Probit Analysis', 2nd Edition. London (Cambridge University Press).
FLINTJER, J. D. AND MEFFERD, R. B.—(1960) *Cancer*, **13**, 172.
GREENE, H. S. N.—(1959) *Proc. Amer. Ass. Cancer Res.*, **3**, 124.
HEWITT, H. B. AND WILSON, C. W.—(1959) *Nature, Lond.*, **183**, 1060.
HOSKINS, J. M., MEYNELL, G. G. AND SANDERS, F. K.—(1956) *Exp. Cell Res.*, **11**, 297.
KAAE, S.—(1953) *Cancer Res.*, **13**, 744.
KAPLAN, H. S. AND MURPHY, E. D.—(1949) *J. nat. Cancer Inst.*, **9**, 407.
MARIE, P. AND CLUNET, J.—(1910) *Bull. Ass. franç. Cancer*, **3**, 19.
MAZUREK, C. AND DUPLAN, J. F.—(1959) *Ibid.*, **46**, 119.
OLCH, P. D., ECK, R. V. AND SMITH, R. R.—(1959) *Cancer*, **11**, 460.
SCHATTEN, W. E.—(1958) *Ibid.*, **11**, 455.
*Idem* AND KRAMER, W. M.—(1958) *Ibid.*, **11**, 460.
TYZZER, E. E.—(1913) *J. med. Res.*, **28**, 309.
WOGLOM, W. H.—(1929) *Cancer Rev.*, **4**, 129.